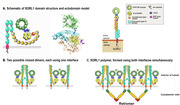# An Introduction to SORL1‐retromer Structure/Function and Its Role in Alzheimer’s Disease

**DOI:** 10.1002/alz.084834

**Published:** 2025-01-03

**Authors:** Gregory A. Petsko

**Affiliations:** ^1^ Harvard Medical School and Brigham & Women’s Hospital, Boston, MA USA

## Abstract

SORL1 (SORLA, LR11) is a large (2214 residue), multi‐domain type 1 integral membrane protein that is the product of the *SORL1* gene. In neurons, where it is highly expressed, SORL1 functions as both a substrate of and a cargo receptor for the retromer multi protein complex that is a master regulator of protein trafficking out of the early endosome. The SORL1‐Vps26b retromer, in particular, is dedicated to the recycling of cell surface proteins, including APP and AMPA receptor subunit GLUA1, back to the plasma membrane. Rare loss‐of‐function alleles of *SORL1*, both truncation and some missense variants are found only in familial Alzheimer’s patients and not in age‐matched normal controls, which has led to the suggestion that *SORL1* is the fourth causal AD gene (after *APP, PSEN1*, and *PSEN2*). More than 500 other variants are known, many of which appear to be pathogenic, with varying penetrance. They encompass all 24 domains of the protein. A crystal structure exists for the VPS10‐10CC region of the protein, which binds peptide cargo such as the Abeta peptide. The extracellular (ectodomain) portion of SORL1 is sometimes shed from the surface of the cell by proteolysis. New data suggests that SORL1 has two potential dimer interfaces, one in the VPS10 domain and one in fibronectin type III (3Fn) region. A model of the entire ectodomain places these two potential interfaces on opposite sides of the protein, implying that if both are used, SORL1 can polymerize to form a network in the interior of the tubular endosome. Such a network would stabilize the tubule from inside, analogous to the way the polymer of retromer arches is thought to stabilize it from outside. Knowledge of the structure and function of SORL1 allows prediction of the possible reasons why certain variants may contribute to the development of Alzheimer’s disease.